# The Diffusion Diaries: Diffusible Iodine-Based Contrast-Enhanced Computed Tomography for Vertebrate Natural History Specimens

**DOI:** 10.1093/iob/obaf014

**Published:** 2025-04-07

**Authors:** J A Gray, E L Stanley, C M Sheehy, Z S Randall, G J Watkins-Colwell, D C Blackburn

**Affiliations:** The University of Texas at Austin, Department of Earth and Planetary Sciences, 2275 Speedway Stop C9000, Austin, TX 78712-1722, USA; Florida Museum of Natural History, University of Florida, 1659 Musuem Road, Gainesville, FL 32606, USA; Florida Museum of Natural History, University of Florida, 1659 Musuem Road, Gainesville, FL 32606, USA; Florida Museum of Natural History, University of Florida, 1659 Musuem Road, Gainesville, FL 32606, USA; Yale Peabody Museum, Division of Vertebrate Zoology, 170–210 Whitney Avenue, New Haven, CT 06511, USA; Florida Museum of Natural History, University of Florida, 1659 Musuem Road, Gainesville, FL 32606, USA

## Abstract

Diffusible iodine-based contrast-enhanced CT (diceCT) is commonly used to create three-dimensional (3D) representations of the soft tissue anatomy of preserved vertebrate specimens. While widely applied, there is currently no documentation of protocols that can be adapted to a morphological and taxonomically broad range of vertebrates. We present the most taxonomically and morphologically broad sampling of diceCT vertebrates, imaged for the openVertebrate Thematic Collections Network. Within this study, we document our methods, outcomes, and observations throughout the preparation, staining, scanning, and data processing steps. Larger specimens take a longer time to stain, but the final staining time depends on the taxon, whether there is dermal and/or bony armor present, and whether any internal structures (e.g., eggs, embryos, large fat deposits) require large amounts of iodine to become fully saturated. We established a scoring system for diceCT-imaged soft tissues that reflects the usefulness of the data. We also provide examples of datasets that demonstrate severe soft tissue damage, incomplete preservation, permanent specimen alteration, and understaining. Finally, we have made all the diceCT datasets produced here freely available to download via the data repository MorphoSource, and hope that our work can serve as a resource for scientists and the public to explore and study vertebrate anatomy.

## Resumen

La tomografía computarizada mejorada con contraste a base de yodo difusible (diceCT) se utiliza comúnmente para crear representaciones tridimensionales (3D) de la anatomía de los tejidos blandos en especímenes de vertebrados preservados. Aunque su aplicación es amplia, actualmente no existe documentación sobre protocolos que puedan adaptarse a una gama morfológica y taxonómica diversa de vertebrados. Acá, presentamos el muestreo más amplio, tanto taxonómica como morfológicamente, de vertebrados mediante diceCT, obtenidos para la red de colecciones temáticas openVertebrate (oVert). En este estudio, documentamos nuestros métodos, resultados y observaciones a lo largo de las etapas de preparación, tinción, escaneo y procesamiento de datos. Los especímenes de mayor tamaño requieren más tiempo para teñirse, pero el tiempo final de tinción depende del taxón, de la presencia de armadura dérmica y/o ósea, y de si algunas estructuras internas (por ejemplo, huevos, embriones, grandes depósitos de grasa) requieren grandes cantidades de yodo para saturarse completamente. Establecimos un sistema de puntuación para los tejidos blandos obtenidos mediante diceCT que refleja la utilidad de los datos. También proporcionamos ejemplos de conjuntos de datos que muestran daños severos en los tejidos blandos, preservación incompleta, alteraciones permanentes en los especímenes y tinción insuficiente. Finalmente, hemos puesto todos los conjuntos de datos de diceCT generados en este estudio a disposición del público y de libre acceso a través del repositorio de datos MorphoSource, con la esperanza de que nuestro trabajo sirva como recurso para que científicos y el público en general exploren y estudien la anatomía de los vertebrados.

## Introduction

Natural history collections are important records of biodiversity, providing the basis for systematic and taxonomic research. They also provide materials for biological studies across a broad range of other disciplines. Many natural history collections contain fluid preserved specimens and access to their unseen internal anatomy is fundamental to many evolutionary studies (e.g., Lillywhite et al. 2011; Hilton et al. 2021). In recent years, digitization initiatives such as the openVertebrate (oVert) Thematic Collections Network (Blackburn et al. 2024) have spearheaded large-scale efforts to digitize museum collections, leading to increased access, limited depreciation of specimens, and broader utilization of resulting data through repositories such as MorphoSource (www.morphosource.org; Boyer et al. 2016). These initiatives foster broader and more advanced research and educational opportunities through enhanced access to collections material (Hedrick et al. 2020). Computed tomography (CT) scanning is now a common method for digitizing fluid-preserved specimens in 3D (Keklikoglou et al. 2019). CT scans can be used to efficiently visualize dense structures within an organism, such as bones and teeth. However, without contrast-enhancing treatment, soft tissue contrast is not typically high enough to distinguish between different types of tissues or different anatomical structures.

Diffusible iodine-based contrast-enhanced CT (diceCT) is a method whereby X-ray-opaque iodine is introduced into the tissues of a specimen via diffusion (Gignac et al. 2016; Metscher 2009). Iodine in the form of triiodide (I_3_^−^) binds to lipids and carbohydrates naturally present in living organisms, which are abundant in the soft-tissue structures of those organisms, including specimens preserved and stored in ethanol (EtOH). Once a specimen is saturated with iodine, soft tissue anatomy will be visible in X-ray images and reconstructed CT scans, which can reveal skeletal, dental, muscular, neural, glandular, epithelial, and special sensory anatomy. Although this technique does chemically alter specimens, diceCT is often significantly less destructive than traditional dissection, as specimens can be “destained” after scanning via leaching or chemical transformation of the staining agent, and the reversibility of diceCT has led to it gaining popularity as a minimally destructive technique (Gignac et al. 2024). In recent years, diceCT has been used in a number of anatomical studies of museum specimens, encompassing a broad range of taxonomic and ontogenetic diversity, and focusing on a range of anatomical structures (e.g., Santana 2018; Camilieri-Asch et al. 2020; Callahan and Crowe-Ridell et al. 2021; Lanzetti and Ekdale 2021; Cleuren et al. 2022; Olroyd 2022; Kolmann et al. 2023; Crowell et al. 2024). The combination of traditional CT and diceCT datasets from the same specimen presents a complete anatomical dataset. Additionally, CT and diceCT can provide access to “natural history by-catch” that includes diet records for both hard and soft-bodied prey (Enge et al. 2023), parasite loads (Ebmer et al. 2022), clutch sizes (Callahan and Crowe Riddell et al. 2021), and stages of reproductive development (Clear et al. 2022; Griffing et al. 2024). The results provide integrative datasets for museum specimens that can be shared widely and used to address questions on form, function, and ecology in evolutionary biology.

Working with natural history specimens to produce soft tissue datasets presents unique challenges. While specimens today are typically fixed in formalin and stored in 70% EtOH (Simmons 2014), variations and deviations in fixing protocols are common, often without detailed records, and the preservation histories of most specimens are unknown. Moreover, specimens may have been stored in freezers before fixation. Although a single freeze-thaw cycle usually does not cause excessive damage to soft tissues, repeated cycles can lead to the formation of ice crystals, resulting in considerable distortion and damage, rendering tissues suboptimal or unusable for comparative studies, particularly affecting delicate neural tissues (Gignac et al. 2016). Despite museums aiming for long-term specimen preservation, it is generally accepted that decades of storage in ethanol can sometimes lead to deterioration of both soft and hard tissues, especially when there are changes in lab protocols over time (Simmons 2014; Leonard et al. 2021). Nevertheless, specimens stored under proper conditions for over a century have been successfully used to generate remarkable CT datasets.

While Lugol's iodine and X-ray imaging are not new tools for anatomical researchers, their combined application holds significant promise for advancing unique anatomical studies. However, the systematic collection of diceCT data from natural history specimens remains limited and vertebrate taxonomic representation has been sparse, particularly among nonmodel organisms. Optimization of diceCT techniques is impeded by a lack of taxon-specific protocols and under-reporting of results, especially negative or poor results. To address this, biologists would greatly benefit from a comprehensive guide outlining best practices for laboratory protocols, data generation, and curation that can be universally applied across diverse taxonomic groups.

**Fig. 1.**
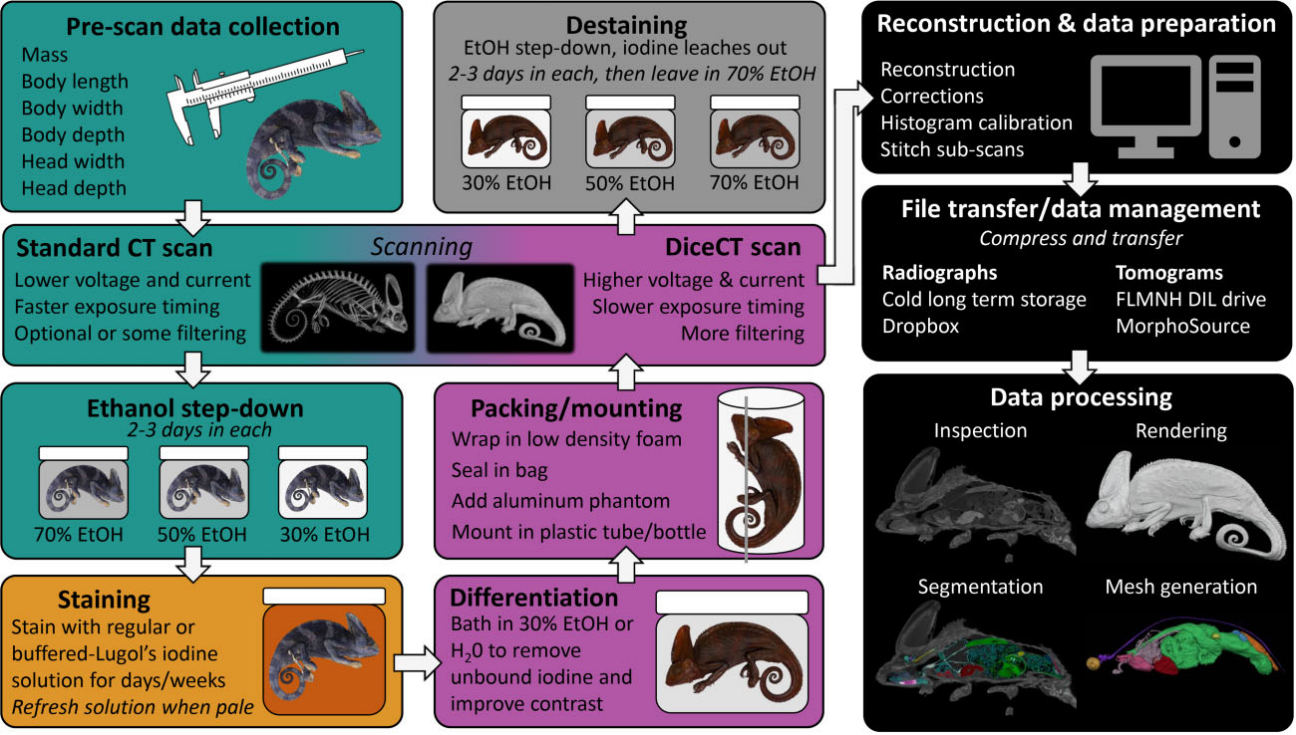
Visualization of the workflow for producing diceCT datasets for vertebrate natural history specimens. Colors demonstrate the stage of the workflow that corresponds with sections in materials and methods: teal = before staining; orange = staining; pink = after staining; gray = destaining; black = data management and processing.

In this study, we create diceCT scans for 204 museum specimens belonging to 179 different vertebrate families including amphibians, reptiles, fishes, and mammals. First, we aim to determine the optimal staining procedure to visualize soft tissues in a taxonomically diverse group of vertebrates, as well as to optimize procedures and estimate staining times for specific taxonomic groups. We use this information to devise an efficient workflow for high-volume scanning of specimens (see Fig. 1), to increase the longevity and usefulness of digital specimens and to minimize damage to physical specimens. Second, we aim to assess the condition of the soft tissues in all imaged specimens to determine the prevalence of soft tissue damage and demonstrate how to detect damaged tissues in diceCT scans. Lastly, we aim to facilitate the future exploration of vertebrate anatomy by making all contrast-enhanced scans produced in this study freely available online for researchers, educators, and the public to access.

## Materials and methods

### Prestaining

#### Specimen selection and prescan data collection

We stained and scanned 204 whole-body specimens from 179 vertebrate families (Table S1), including representatives from Amphibia, Reptilia, Mammalia, Chondrichthyes, and Osteichthyes. Specimens encompassed a range of body sizes, with body length (measured in snout–vent length for amphibians and reptiles, snout to caudal base in fishes, and total length excluding tail for mammals) ranging from 12 to 998 mm and mass ranging from 0.1 gm (*Ptilichthys goodei* VIMS:40875) to 924 gm (*Calyptocephalella gayi*, UF: herp:64704). Specimens were primarily from the collections at the Florida Museum of Natural History (University of Florida; UF), and supplemented by loans from the Yale Peabody Museum (YPM), California Academy of Sciences, Field Museum of Natural History, Carnegie Museum, University of California at Berkeley's Museum of Vertebrate Zoology, and the Virginia Institute of Marine Science (VIMS). Since specimen age has been suggested to have an effect on the quality of soft tissue, we prioritized more recently collected specimens. Overall, we sampled specimens collected from the years 1924–2022. When possible, we also considered the position in which specimens were fixed, which can sometimes allow for higher resolution scans and decreased scan time. This included avoiding coiled specimens (e.g., snakes preserved in a spiral shape) to avoid the need for X-rays to penetrate through multiple layers of the body, and choosing individuals that were easier to manipulate into appropriate positions. We also avoided specimens with dissections or other cut surfaces, because cut surfaces are prone to faster staining and overstaining, potentially leading to irregular stain distributions. We measured and recorded the body dimensions of each specimen including body length, snout–vent length, snout–caudal base length, tail length (where applicable, and caudal fin length for fish), head width, head depth, body width, body depth, and mass (see Table S1).

#### Skeletal CT scans

All scans, both skeletal and contrast-enhanced, were conducted at the Nanoscale Research Facility at the University of Florida, using a GE Phoenix v|tome|x m. All specimens were scanned prior to staining to acquire skeletal datasets. Standard CT scans were generated using a lower current and voltage, with a faster exposure time and less filtering, compared to diceCT scans. We conducted skeletal scans before staining at a range of 50–170 kilovolts (kV, voltage) and 50–200 microamperes (µA, amperage), with 200–250 millisecond exposure (ms), with 1000–2200 projections (dependent on image width, usually 1.2× the width of images in pixels), filtering depending on specimen external density (typically 0.1 mm Copper or 0.3 mm Aluminum, and sometimes 0.5–1 mm Copper for large specimens or those with many overlapping body parts in the field of view [e.g., large coiled snakes]), with 3× frame averaging and one frame skip per rotation. For wide specimens, two scans were conducted, one of the whole specimen and a higher-resolution scan of the head. Each specimen scanned also included a separately attached material of known density, a piece of 0.5 mm gauge aluminum wire. The density of this aluminum wire (2.7 g/cm^3^) is comparable to the density of cortical bone (see Aerssens et al. 1998). The artifacts produced from the aluminum wire were minimal, although slightly more noticeable with lower beam energies, and had little to no effect on the anatomical data. During reconstruction, we used the aluminum wire to calibrate the histogram, spreading the grayscale values (GSVs) appropriately across the dataset. This aluminum wire could also be used to detect decalcification of bone in the specimens. While useful, the inclusion of aluminum wire is not critical to producing high quality datasets, and users may use any structures within the datasets to calibrate the histogram to suit their research. Suitable aluminum wire can be purchased from many scientific supply companies (e.g., Thermofisher product 042238.H2). After histogram calibration, reconstruction was considered complete, and the data were transferred.

#### Ethanol step-down

To minimize osmotic shock to ethanol-preserved specimens stained in a water-based solution, we stepped down the concentration of ethanol in which specimens were stored before staining. We stepped down the EtOH concentration from 70%, to 50%, to 30%, leaving them for 2–3 days in each solution, before finally putting them in a staining solution. The ethanol downgrade may lessen the effects of osmotic shock of moving specimens from alcohol to the water-based Lugol's iodine solution, and vice versa (pers. obs. P Gignac; Simmons 2014).

### Staining

After stepping down the EtOH concentration, all specimens were stained using Lugol's iodine (see Supplemental File 1 for wet lab protocol). Specimens stained during the first year of the project (before December 2021) were stained using standard Lugol's iodine (I₂ + KI + H₂O). Following the publication of the Dawood et al. (2021) method for reducing shrinkage while using Lugol's iodine, we transitioned to using buffered Lugol's iodine. The buffered Lugol's iodine was made to the same concentration of iodine as the standard solution, with the addition of a 2× Sorensen's buffer. There was no noticeable difference in staining duration between the two different solutions. For most specimens, we used a 1.25% w/v Lugol's solution for both buffered and unbuffered solutions. In the following two cases, we deviated from this concentration:

- **Crocodilians**: we stained three crocodilian specimens. These were considerably larger (215–415 gm) than other specimens and were heavily armored with osteoderms. We therefore increased the iodine concentration to 3.75%, which is still within the recommended range of concentrations for Lugol's iodine solutions (Gignac et al. 2016).
- **Turtles**: we stained eight turtles. To begin, two of these were stored in a 1.25% Lugol's iodine solution, one of which was given daily iodine injections of the same 1.25% solution. The results from these preliminary datasets (Gray et al. 2023) provided evidence that allowed us to refine the staining technique for turtles. For the remaining six turtles, we used a 1.25% Lugol's iodine bath with daily injections of 2.5% Lugol's iodine. The increase in concentration of the injected solution was implemented to facilitate faster diffusion and therefore shorter staining times for the remaining turtles.

**Fig. 2.**
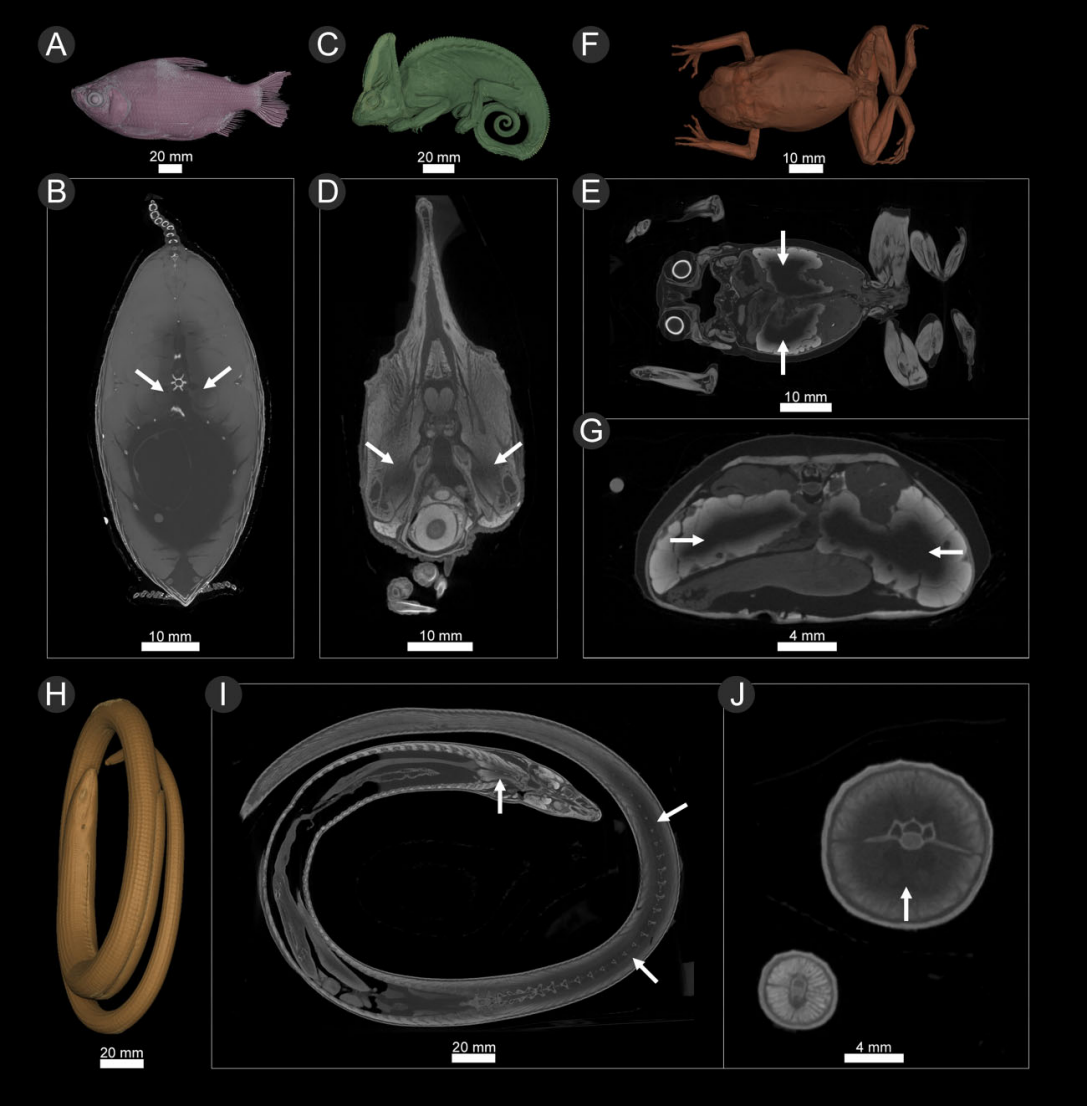
Three dimensional renderings and tomograms from “quick scans” of under-stained specimens. Arrows show regions where a diffusion boundary was observed, indicating specimens are under-stained and require longer in staining solution. (**A–B**) *Curimata cyprinoides* (UF: fish:32308) 3D-rendering of whole body (**A**) and tomogram showing transverse cross section through body. (**C–D**) *Chamaeleo calyptratus* (UF: herp:191369) 3D-rendering of whole body (**C**) and tomogram showing transverse cross section through the head. (**F–G**) *Leptopelis notatus* (UF:herp:180845) 3D-rendering of whole body (**F**) and tomograms showing longitudinal cross section through the entire body, and transverse cross section through the body. (**H–J**) *Ophisaurus attenuatus* (UF:herp:84436) 3D rendering of entire body (**H**) and tomograms showing longitudinal cross section through entire body (**I**) and transverse cross section through body (**J**).

As staining progressed, the staining solutions became paler as the iodine was depleted. To maintain concentrations that facilitate diffusion, we monitored the appearance of staining solutions throughout the staining process and replaced staining baths with fresh solution when it became considerably more transparent. Most of the time, adequately stained specimens took on a dark amber or brown color, often obscuring external color patterns visible prior to staining. Specimens with incomplete staining can sometimes look paler than fully stained specimens and light red or yellow in color, although it is typically not possible to determine if a specimen is fully stained without a test scan or via dissection. We noted that some specimens that were completely saturated with iodine could appear paler (i.e., closer to the original color) on their exterior than other fully stained specimens if their iodine baths had become pale and depleted of iodine. The length of time that a specimen took to stain was roughly inferred from its body size. When we estimated that staining was nearing completion, we confirmed this by conducting a 3–5 min “quick scan” (low resolution CT scan with no frame averaging and a low number of projections, as set by the “quick scan” option on the GE phoenix v|tome|x, also described by Callahan and Crowe-Riddell et al. 2021) to reveal staining progress and allow us to adjust staining times accordingly. Quick scans of under-stained specimens revealed a diffusion boundary with noticeably darker regions in the thickest parts of the animal where iodine had yet to penetrate (Fig. 2). Optimally stained specimens resulted in 2D tomography slices with consistent contrast across all tissues. If a diffusion boundary was detected (Fig. 2), the specimen was returned to a fresh iodine bath.

### Poststaining

#### Differentiation

After staining, all specimens were transferred to a 30% EtOH bath (for larger specimens e.g., >50 g) or a water bath (for smaller specimens e.g., <50 g). This served to wash away unbound iodine from the outside of the specimens, and to slightly de-stain any overstained extremities. Very small specimens (i.e., body length <50 mm) were bathed in the EtOH for no longer than 30 min. Medium-sized and larger specimens were bathed for approximately 60 min.

#### Packing and mounting

Stained specimens were wrapped with thin foam sheets covering the external surfaces of the body and separating different parts of the body from one another if necessary (e.g., coils of a snake, bat wings). To keep body parts in place, we arranged packing peanuts (low density and not visible in the scans) around the head or limbs. Specimens and surrounding low-density materials were placed inside appropriately sized zip-lock bags and the bag was sealed with as little air inside as possible. The empty portion of the zip-lock bag was then wrapped around the specimen, and we added a piece of aluminum wire close to the specimen, along the long axis (not touching the specimen and placed outside of the zip-lock bag), that would appear in all cross-sections and could be used later for histogram calibration. Specimens were then inserted vertically into open-ended polyethylene cylinders or plastic bottles with the base cut-off (which could then be clamped onto the mounting platform using the neck of the bottle). Where possible, and to facilitate higher quality 3D-renderings, we positioned the specimen to lie above the bottom end of the bag, so that any pooling liquid would not surround the lowest components of the specimen. We found that the optimal positioning for small-to-medium sized specimens was placing the head upwards to minimize the chance of staining solution pooling around the head during the high-resolution head scans. Low-density materials including pieces of ethafoam, packing peanuts, and bubble wrap were used to firmly fill the spaces between the sealed specimen and the plastic cylinder, ensuring the specimen was stable and minimizing movement during the scan. If specimens protruded partially out of the end of the plastic cylinder, loose parts were held in place with packing tape. Since any movement was likely to occur soon after packing, we left the packed specimen still for at least 30 min, preferably over 60 min, and sometimes overnight for complicated mounting conditions, before scanning.

#### DiceCT scanning

Specimens stained with iodine are more radio-dense and therefore require higher beam energies than nonstained standard CT scans. To produce high-quality diceCT scans, we used higher beam energy than was used for skeletal scans, and used thicker filters. We maintained high signal-to-noise ratios by increasing X-ray intensity (voltage and amperage) and longer detector exposure times. Higher X-ray tube currents increase the number of X-rays produced and higher (i.e., slower) exposure timing allows for the X-ray detector to collect more X-rays passing through the specimen, and multiframe averaging then collects X-ray density data several times for the specimen in one rotation position, then averages these samples. This approach collects a substantial amount of image data, producing scans with sharper tissue boundaries and smoother gray-scale value transitions. We conducted diceCT scans at a range of 50–210 kilovolts (kV, voltage) and 65–300 microamperes (µA, amperage), with 200–500 millisecond exposure (ms), with 1000–2400 projections (dependent on image width), filtering (usually either Al 0.3 mm, Cu 0.1 m, or Cu 0.5 mm), and 3× frame averaging with one skipped frame per rotation. To ensure maximum resolution, full body scans were generated in multiple parts along the length of the body, with as many as six partial scans that were later stitched together into a single dataset. We provide a complete list of scanning parameters for all 252 diceCT scans in Table S2. This list was generated using the individual GE Phoenix acquisition metadata files (PCA) for each scan, with the aid of an R script written by ELS. The R script can be found on GitHub (https://github.com/drScanley/CTMetadataExtractor).

### Reconstruction and data preparation

#### Reconstruction

We reconstructed raw tomography projections using GE Datos R software (General Electric, version 2.8.2), which generated cross-sectional images in volume file format (.vol). To calibrate the histogram and stitch sub-scans together, we imported the reconstructed volumes into *Volume Graphics (VG) Studio Max*, using versions 5-2024 throughout the years the scans were generated.

#### Histogram calibration

We calibrated the histogram with 0–65,536 GSVs using air in the space surrounding the specimens and the aluminum (Al) phantom. Typically, we mapped the density of air to 10,000 GSV, and the phantom to either 30,000, 40,000, or 50,000 GSV, depending on the distribution of GSVs in a scan. For scans containing comparatively more high density material, we calibrated Al to 30,000 (to achieve a more even spread of the dense material across the histogram), for scans with comparatively less high density material, we calibrated Al to 50,000 (to achieve a more even spread of the less dense material across the histogram). This ensured that the GSVs for soft tissues were evenly distributed across the histogram and allowed for easier stitching, segmentation, and processing. For calibrating subscans that were to be stitched together, we used the regions of overlap between two scans, choosing an equivalent cross-section from each scan to use for histogram calibration.

#### Sub-scan stitching

To stitch sub-scans together using *VGStudio Max*, volumes were first roughly aligned by eye using the “surface determination function” and “simple alignment” tool, then aligned with a Gaussian best fit using the “best-fit alignment” tool. Once aligned, sub-scan volumes were then merged using the “max” operation in the “merge and resample” tool (which retains the higher gray-value of overlapping voxels). Once stitching was complete, we saved the full scan as a TIFF image stack. Since the histograms were calibrated appropriately prior to stitching, no further image processing was needed to enhance the contrast.

### Destaining

After specimens were successfully scanned, an ethanol destaining protocol was used (see Gignac and Kley 2018). This protocol excluded the use of additional solvents (e.g., sodium thiosulfate as in Schmidbaur et al. 2015; Gignac et al. 2016), which resulted in highly variable destaining duration depending on specimen size. To de-stain specimens, we used ethanol baths to leach the iodine from the tissue. After scanning, specimens were placed in 30% ethanol, followed by 50% ethanol, and finally 70% ethanol, with 2–3 days between each step-up. This ethanol step-up served to reduce osmotic shock to the specimen, and iodine began to leach out during this process, as indicated by ethanol becoming more yellow/brown in color as time progressed. After that, the 70% ethanol was refreshed regularly until it became clear and colorless, and remained so after several weeks. Specimens were then returned to their respective institutions to be integrated back into collections.

### Scan evaluation

**Fig. 3.**
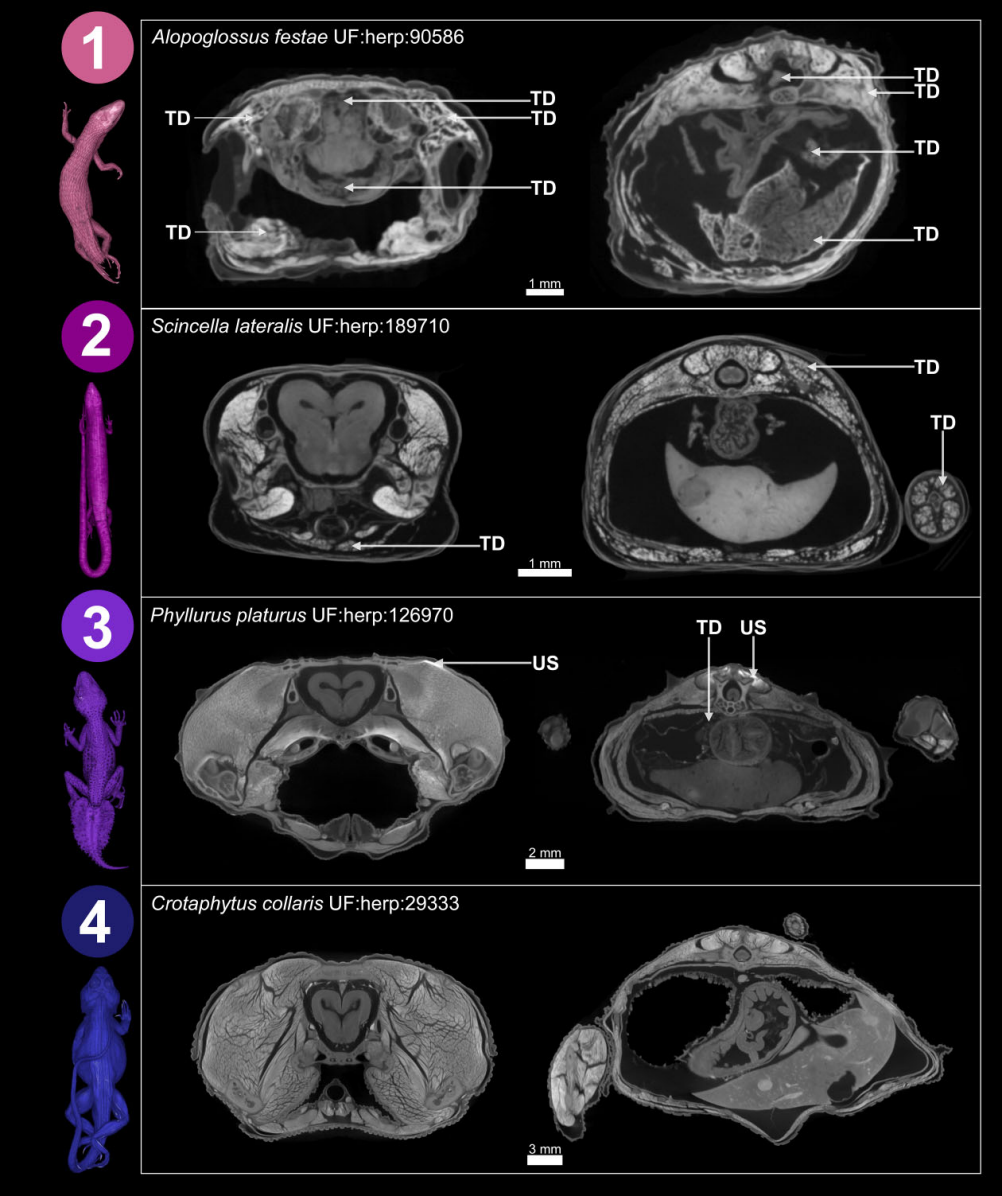
Tomograms from four different diceCT datasets that demonstrate the Tissue Condition Score (TCS) used to assess tissue quality in this study. TCS are as follows: 1 = very low quality, with widespread damage and very little or no usable data imaged; 2 = somewhat low quality, with substantial tissue damage but some usable data; 3 = mostly good quality, with some tissue damage detected but most data usable; 4 = very good quality, no tissue damage detected and all data usable. For each TCS, we show a cross-section through the head, capturing the brain (optic tectum), structures inside the mouth, jaw muscles and integument (left) and a cross section through the torso, capturing the liver, gut, lungs, spinal cord, body musculature, and integument. Three-dimensional renderings of whole specimens are shown on the left-hand side. Arrows indicate areas with visible tissue damage (TD) and uneven stain distribution (US).

We examined each scan to assess the condition of the soft tissue. J.A.G. developed a simple scoring system to evaluate the overall condition of the soft tissues for each specimen that essentially reflects the usability of each diceCT scan. Tissue Condition Scores (TCSs) are as follows: 1 = very low quality, with widespread damage and very little or no usable anatomy detected; 2 = somewhat low quality, with substantial tissue damage but some usable anatomy; 3 = mostly good quality, with some tissue damage detected but most anatomy usable; 4 = very good quality, no tissue damage detected and all anatomy usable (see Fig. 3 for examples and Supplemental File 2 for guidance on scoring). We noted which specimens had been dissected or had some tissues removed. We also examined scans and recorded the sex, presence of eggs, stomach contents, parasites, and other notable features.

### Staining duration evaluation

**Fig. 4.**
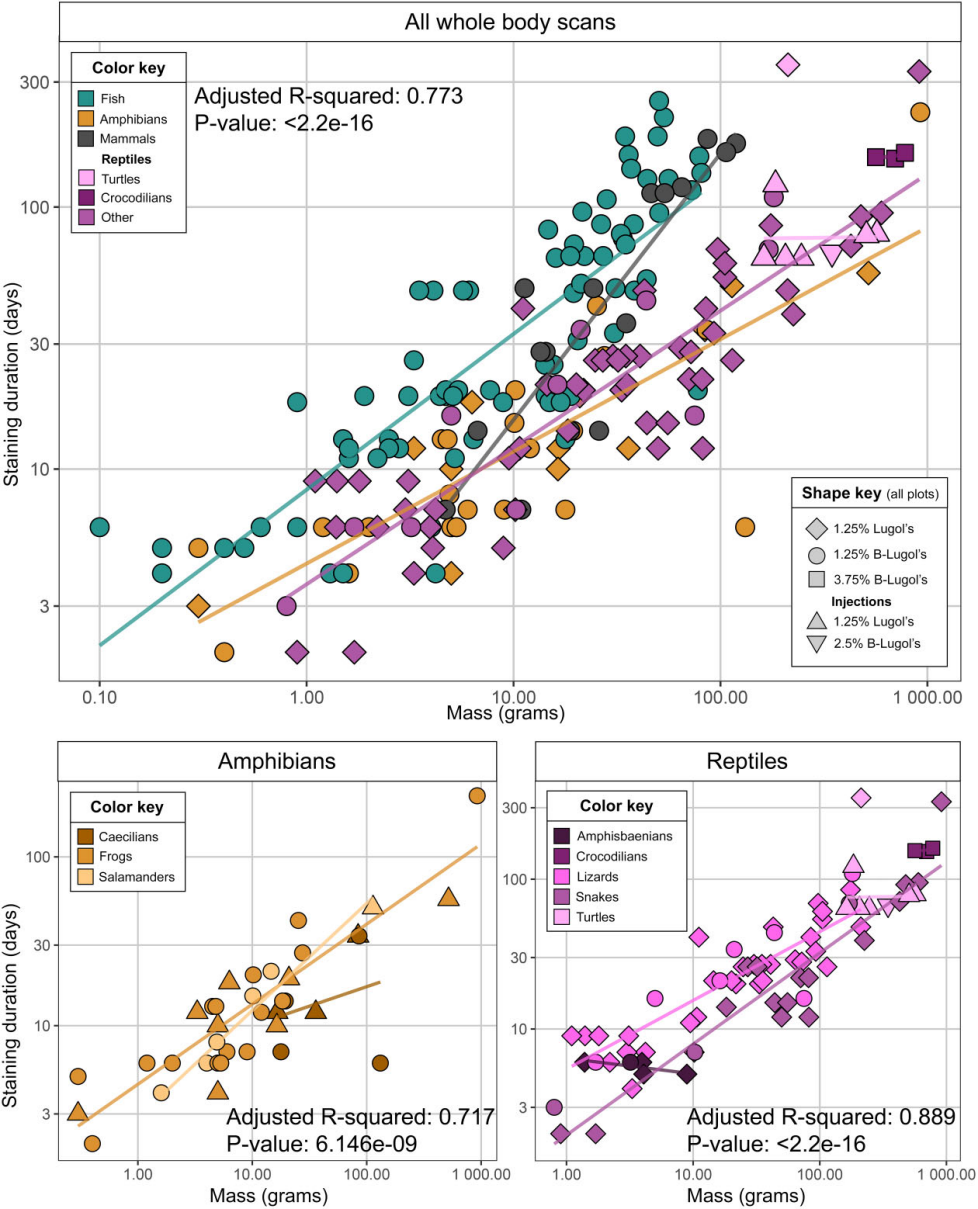
Plots showing mass on the X axis and time taken for specimens to be fully stained on the Y axis, both log10 transformed. Points are colored by vertebrate group, including fishes amphibians, mammals, and reptiles. In the main (top) plot, reptiles that underwent a different staining treatment (turtles and crocodilians) are highlighted in different colors. The shape of the points shows the staining treatment used for that individual. Bottom plots show amphibians (left) and reptiles (right), highlighting different taxonomic groups.

Although we collected several parameters for body dimensions, due to the broad diversity in body shape in our dataset, body mass was the best overall predictor of staining durations for vertebrates. To assess how body mass correlates with staining duration, we used RStudio (version 2024.12.1) to fit a linear model (lm(log10(days to stain) ∼ log10(mass) * group) and to acquire adjusted R-squared and *P*-values. We chose to log transform the data due to the high number of smaller specimens in the dataset, compared with larger specimens. We first fit the linear model for all specimens in the dataset, and then produced separate plots and linear models for amphibians and reptiles, since these taxonomic groups included extreme variation in body shape, and some differences in stain treatments (see Fig. 4). We provide all body dimension measurements (see Table S1) collected for specimens included in this study, so that researchers may use this information to consider staining times for their particular taxon of interest.

### Data storage, management, and processing

#### Radiograph and metadata storage

After the reconstruction process, we compressed the raw scan data as a ZIP file, which includes the metadata files and radiographs, and archived them on cold storage. As the staining process involves a significant amount of time, effort, and chemical stress on the specimen, these datasets are unlikely to be re-creatable with subsequent scanning. The retention of diceCT raw files is therefore more important than retaining the raw files from unstained CT scans, which can be easily reproduced through a new imaging event. While these data are not necessarily needed for further processing or research, they are archived at the University of Florida in case the tomogram files become corrupted, a researcher later finds the need for a reconstruction using different parameters, or novel reconstruction methods are developed that can improve resolution and/or scan quality (see e.g., the deep learning neural network approaches in ZEISS Advanced Reconstruction Toolbox).

#### Reconstructed tomogram storage

The reconstructed image stacks in TIFF format were compressed and uploaded to MorphoSource with links to the specimens’ collection object and collecting event records imported from iDigBio (iDigBio.org). Contrast-enhanced scans were added to respective project pages on MorphoSource. Because any later derivative postprocessing by the authors, including rendering, segmentation, and 3D model generation, would be done in the Florida Museum of Natural History's Digital Imaging Division, a copy of these files was also moved to the division's network storage drive.

Morphosource project pages created for each group:

Fish: https://www.morphosource.org/projects/000598946

Amphibians: https://www.morphosource.org/projects/000600196

Reptiles: https://www.morphosource.org/projects/000602028

Mammals: https://www.morphosource.org/projects/000604886

#### Postprocessing

Further processing of the 3D volumes, including visualization, rendering, segmentation, and mesh generation, was conducted in the Florida Museum of Natural History's Digital Imaging Division's 3D processing laboratory, using *VGStudio Max* (Volume Graphics, versions 3.4, 3.5,2022.1,2022.2,2022.3,2022.4,2023.1,2023.2,2023.3,2023.4,2024.1, and 2024.3). To create the figures in this study, we used a combination of different processing tools including the paintbrush, region growing, open/close, erode/dilate, clean ROI, split ROI, and edge refinement.

## Results and observations

Typically, larger specimens took longer to become fully saturated with iodine (Fig. 4). However, this varies across datasets and several factors lengthen staining times. We provide separate linear equations for fishes, amphibians, reptiles, crocodilians, turtles, and mammals that provide a starting point for estimating staining time (Table 1). Due to the variation in the dataset, these estimates should be considered simply as a starting point. Staining times varied due to body shape, abnormalities in the underlying anatomy, reproductive phase (e.g., eggs or developing fetuses can lengthen staining time), presence of any cuts or dissection and presence of bony or integumental armor. For a given size, amphibians and reptiles typically stained the fastest. Fish and mammals of the same mass stained more slowly, perhaps due to the higher relative muscle mass in these taxa (Bishop et al. 2021). Mammals stained the slowest, with a steeper increase in staining duration with an increase in body size.

**Table 1.** Predictive equations for time taken to stain mammals, fish, amphibians, crocodilians, turtles, and all other reptiles

Group	Stain concentration	Intercept	Slope	Equation	Adjusted R-squared	Days to stain 18g (median mass) specimen
Mammals	1.25%	0.172	1.016	10^(0.172 + 1.016*mass)	0.7599	28
Fish	1.25%	0.921	0.595	10^(0.921 + 0.595*mass)	0.6929	47
Amphibians	1.25%	0.639	0.428	10^(0.639 + 0.428*mass)	0.7167	16
Crocodilians	3.75%	1.93	0.093	10^(1.93 + 0.093*mass)	0.6074	111
Turtles (excluding *Megacephalus* as outlier)	1.25% bath + 2.5% injections	1.363	0.19	10^(1.858 + 0.010*mass)	0.9048	74
Reptiles	1.25%	0.56	0.523	10^(0.560 + 0.523*mass)	0.7831	15

Staining observations made from test scans about structures that govern staining times:

- **Lizards**: the head typically took the longest to stain, as the staining agents took some time to move through the jaw musculature and into the region containing the hindbrain. The exceptions include lizards in the families Varanidae (*Varanus prasinus*) and Teiidae (*Amieva amieva*), where the large hip and thigh muscles took the longest. Heavily armored lizards presented a challenge, with lizards such as *Gerrhosaurus, Ophisaurus*, and *Ctenosaura* taking longer to stain than other lizards of a similar size.
- **Amphisbaenians**: stain uptake was even throughout the length of the body.
- **Snakes**: the head was often stained before the rest of the body, and in some cases we were able to generate high-resolution scans of the head before the rest of the body was fully saturated with iodine. Many snakes scanned had large amounts of fat storage (e.g., *Xenopeltis, Eryx*), and this appeared to lengthen the time taken to fully stain.
- **Turtles**: for most turtles, the hip muscles were the last remaining structure to be fully stained. The one deviation from this pattern was *Megacephalus*, whose exceptionally large head and associated musculature took the longest to stain. Since we only stained one turtle with a head this size, future studies must consider this if staining comparable specimens (we also excluded *Megacephalus* from the predictive equations in Table 1, as it was inhibiting the ability to predict staining times for turtles with more average-sized heads).
- **Crocodilians**: the head (jaw muscles and brain) and hips took the longest time to stain.
- **Frogs and salamanders**: frogs and salamanders stain relatively quickly. Of the 24 frogs scanned, nine had eggs present. Of the seven salamanders scanned, four had eggs present. In all specimens with eggs present, the eggs were the last structures to be fully stained. In frogs without eggs present, either the brain or the hips and thighs were the last remaining structures to be stained (e.g., for frogs known for their large leg muscles such as *Pseudis*). For salamanders without eggs, the head and hindbrain were the last remaining structures to be stained.
- **Caecilians**: stain uptake was even throughout the length of the body.
- **Mammals**: the neck and shoulders, and brain were typically the last structures to remain under-stained. The speed of staining seemed to be decelerated by large amounts of muscle mass around the shoulders and an almost completely enclosed braincase.
- **Fish**: in almost every case, the muscular mid-body of a fish was the last thing to remain under-stained. Many fish bodies are effectively made up of a large “tube” of muscle, including large masses of epaxial and hypaxial musculature surrounding the internal organs of the abdominal cavity, and that appears to contribute to their long staining times.

Based on these observations and the high degree of variation in stain times, we recommend using our taxon-specific predictive equations as an estimate of staining duration, while keeping in mind that the eventual time taken to stain a specimen may be shorter or longer than predicted.

**Fig. 5.**
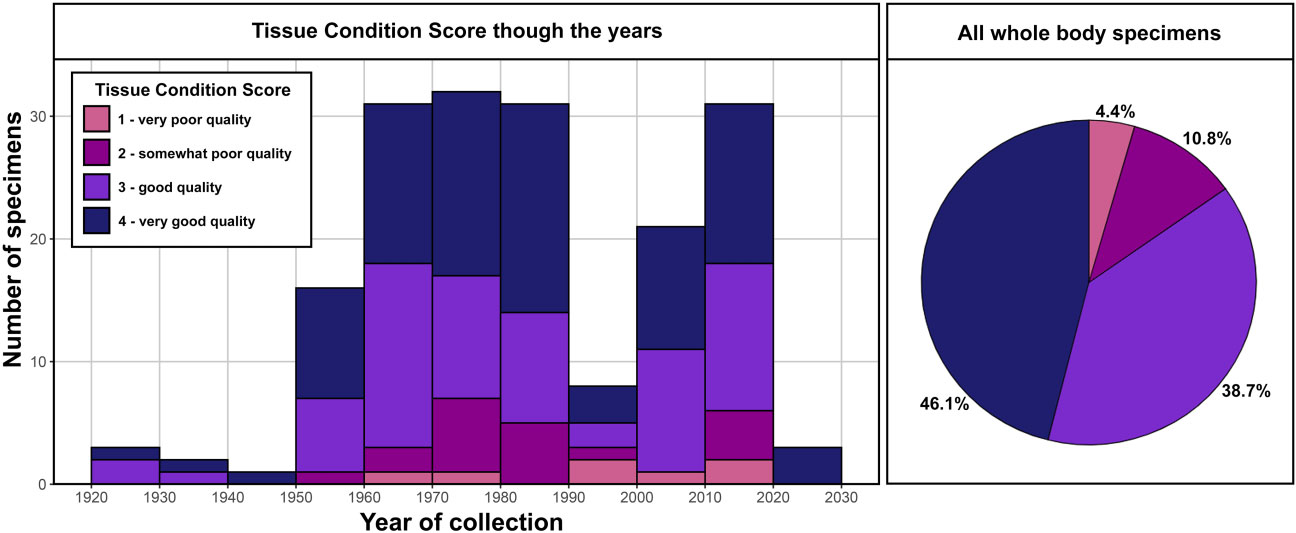
Tissue Condition Scores (TCS) for all specimens in this study. The histogram (left) shows collection years of specimens in the dataset and TCS throughout the decades. The pie-chart (right) shows overall TCS.

The quality of tissues as reflected by the TCS varied widely among the specimens included in this study (Fig. 5). The majority of specimens produced usable scans, with most specimens scoring a 3 (38.7% of specimens) or 4 (46.1% of specimens). Some specimens produced considerably poor quality scans but with some usable components (score = 2, 10.8% of specimens), and a small number produced essentially unusable scans with a score of 1 (4.4% of specimens). Interestingly, the amphibian specimens included in this study have consistently high TCS, with all scoring a 3 or 4 (all TCS scores available in Table S1).

When we observe the TCS in the context of collection dates (Fig. 5), older specimens do not necessarily have poorer quality tissues. There is no apparent association between collection date and TCS. In fact, we noted that some of the oldest specimens included in this study produced high-quality soft tissue scans, and more recent collections have a higher proportion of poorer quality tissues. This could be an artifact of our sampling or of differences in preservation practices over the years. Overall, these results strongly suggest that more recent specimen collections do not necessarily produce better scans, and specimens that are correctly fixed and stored can be used for diceCT scanning decades after they were collected.

### Utilization of high-quality diceCT scans

**Fig. 6.**
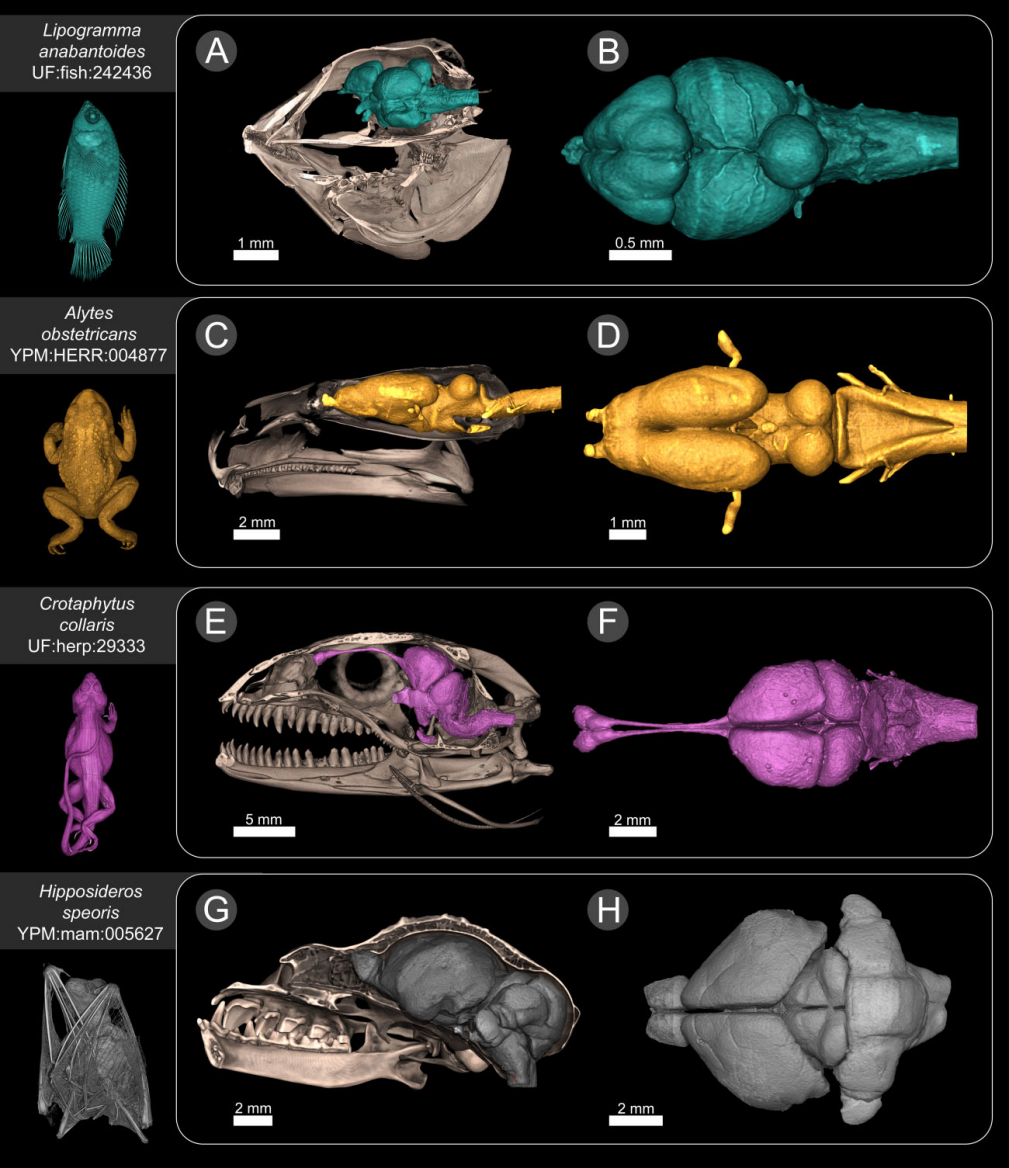
Three-dimensional renderings of the brains of a dusky basslet (*Lipogramma anabantoides* UF:fish:242436 [**A** and **B**]), a midwife toad (*Alytes obstetricans* YPM:HERR:004877 [**C** and **D**]), a collared lizard (*Crotaphytus collaris* UF:herp:29333 [**E** and **F**]), and a Schneider's leaf-nosed bat (*Hipposideros speoris* YPM:MAM:005627 [**G** and **H**]). In A, C, E, and G, brains are shown in the lateral view with bisected skull showing brain placement inside the head. In B, D, F, and H, brains are shown in dorsal view. A 3D rendering of the whole specimen is shown on the left.

**Fig. 7.**
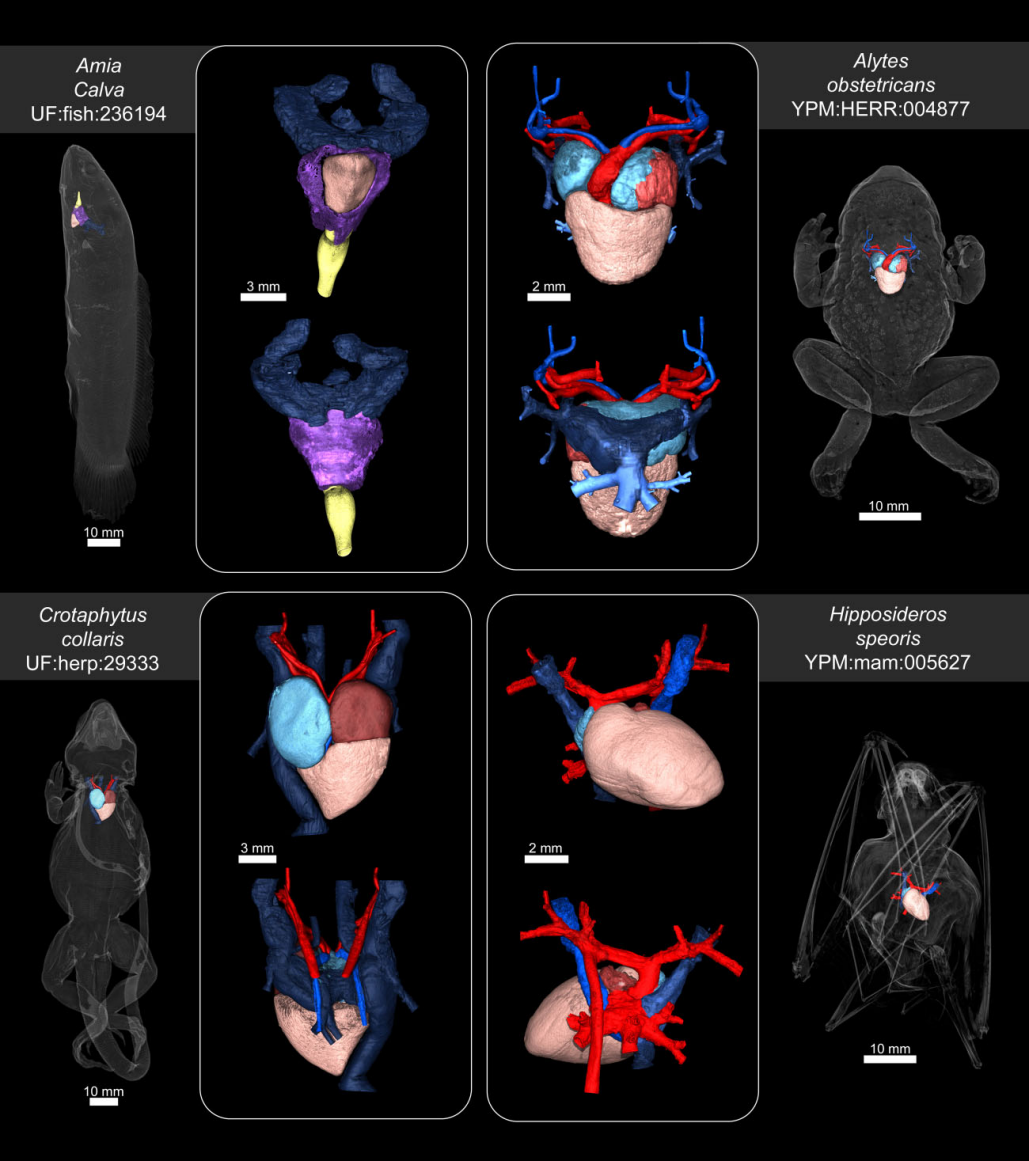
Three-dimensional rendering of the hearts of bowfin (*Amia calva* UF:fish:236194 [top left]), a midwife toad (*Alytes obstetricans* YPM:HERR:004877 [top right]), a collared lizard (*Crotaphytus collaris* UF:herp:29333 [bottom left]), and a Schneider's leaf-nosed bat (*Hipposideros speoris* YPM:MAM:005627[bottom right]), transparent renderings of whole animal with heart place inside are shown on the outside of the image. Anatomical structures are rendered in different colors to demonstrate the variation in heart anatomy across different vertebrates.

We were able to provide examples of several different anatomical structures that can be easily isolated from diceCT scans. We isolated the brains of a dusky basslet (*Lipogramma anabantoides* UF:fish:242436), a midwife toad (*Alytes obstetricans* YPM:HERR:004877), a collared lizard (*Crotaphytus collaris* UF:herp:29333), and a Schneider's leaf-nosed bat (*Hipposideros speoris* YPM:MAM:005627) (see Fig. 6). Skulls of specimens were digitally extracted from the corresponding skeletal scan and aligned with the diceCT scan, so that correct brain placement inside the skull could be visualized. We also isolated the hearts of a bowfin (*Amia calva* UF: fish:236194), a midwife toad (*Alytes obstetricans* YPM: HERR:004877), a collared lizard (*Crotaphytus collaris* UF: herp:29333), and a Schneider's leaf-nosed bat (*Hipposideros speoris* YPM:MAM:005627) (see Fig. 7). Different anatomical regions of the heart were rendered in different colors to illustrate the variation in anatomy across different vertebrate groups. The isolation and rendering of brains and hearts demonstrates the utility of diceCT scans in comparative anatomy studies, and morphological characterization of soft tissue structures *in situ*.

### Poor quality soft tissues

**Fig. 8.**
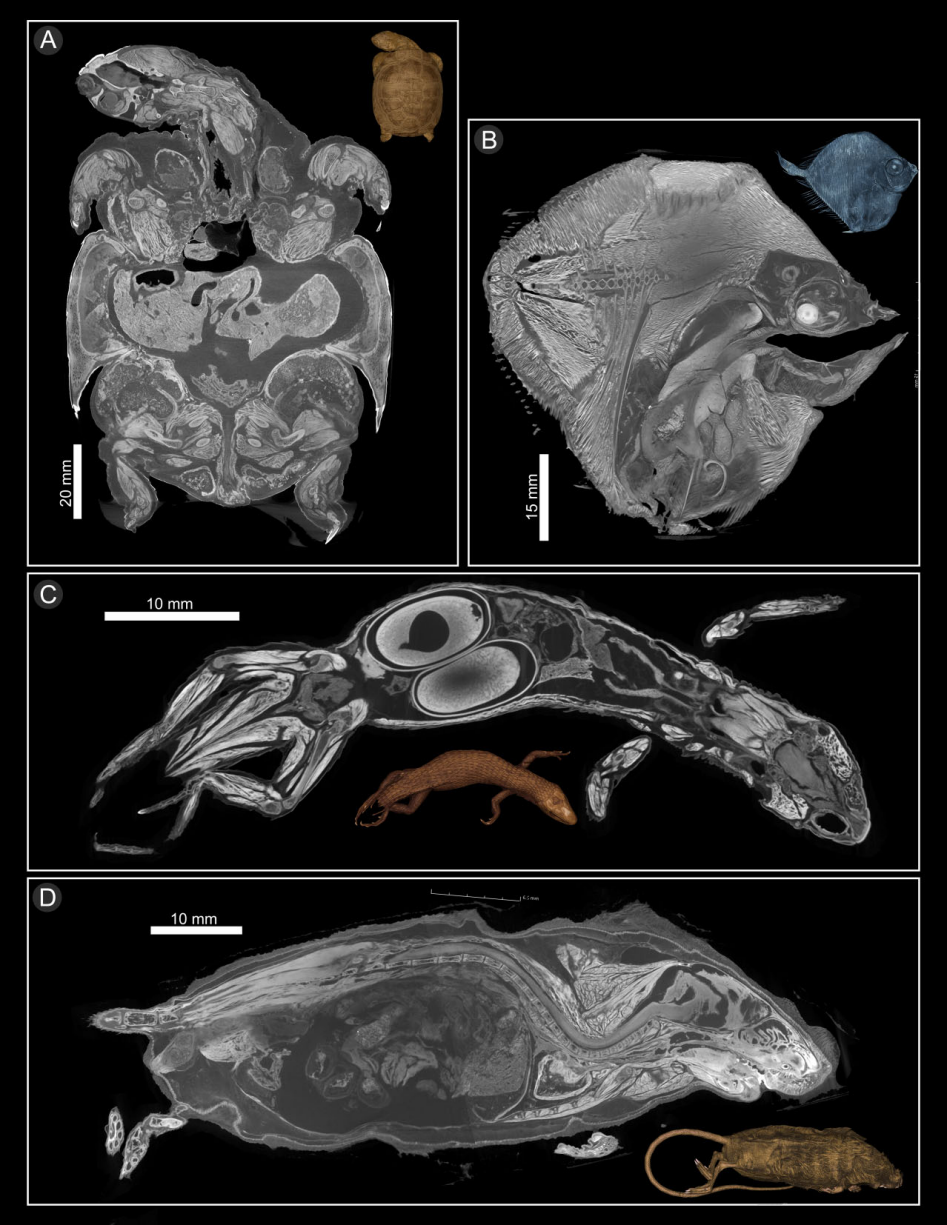
DiceCT cross-sections from specimens with widespread tissue damage and a tissue condition score (TCS) of 1, including a 3D rendering of the whole specimen. (**A**) *Pelomedusa subrufa* UF:herp:85211. (**B**) *Xenolepidichthys dalgleishi* YPM:ICH:011455. (**C**) *Alopoglossus festae* UF:herp:90586. (**D**) *Zapus hudsonius* YPM:MAM:005659.

**Fig. 9.**
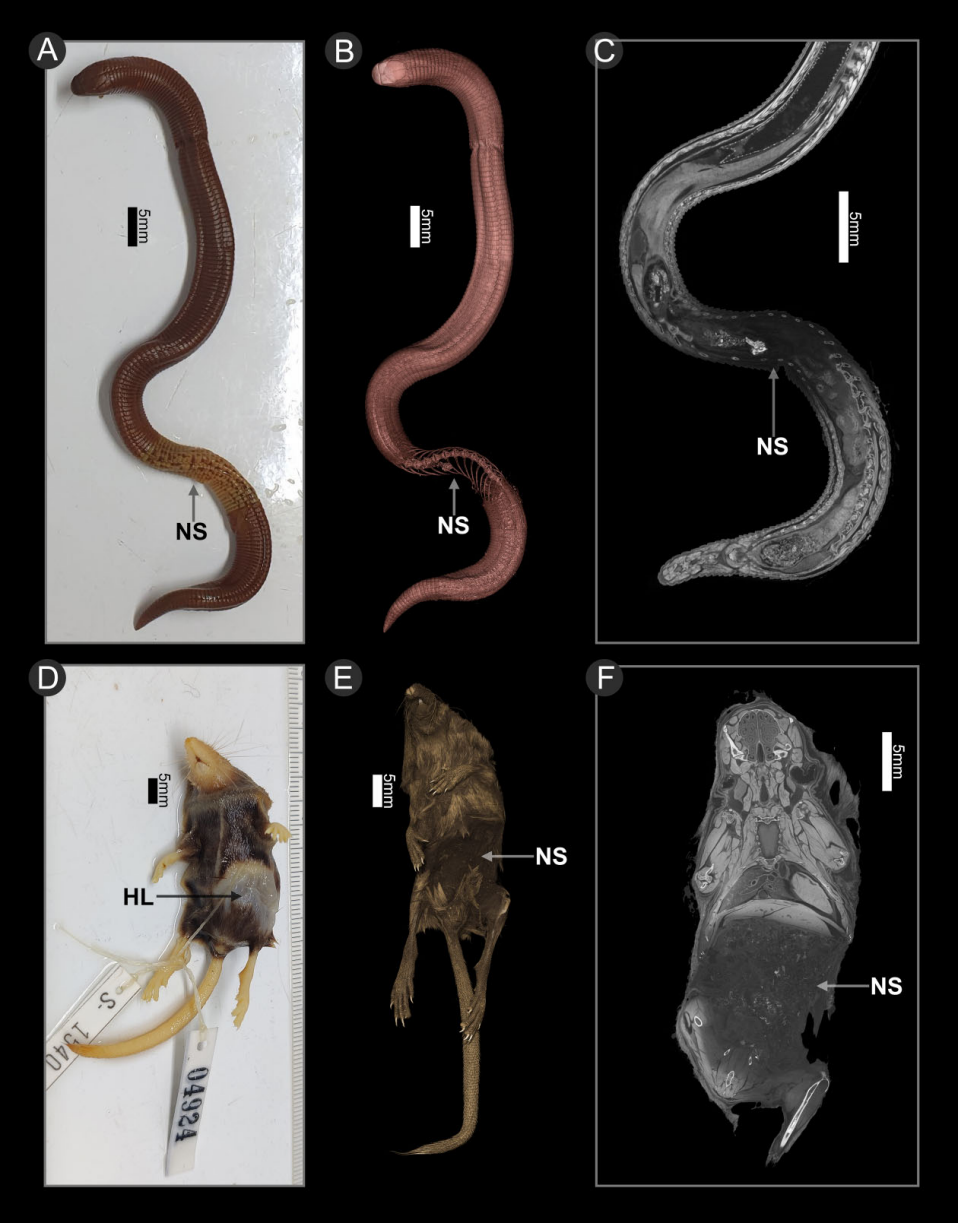
Examples of specimens that have been incompletely fixed. Arrows demonstrate areas that are not stained (NS), or show hair loss (HL). Top panels (**A–C**) show Zarudny's worm lizard (*Diplometopon zarudnyi* UF:herp:68567) with an incompletely fixed section near the posterior end of the body. The specimen's appearance after staining (**A**) shows a section of the body that has not taken up any iodine solution, and the 3D rendering (**B**) and cross-section through the body (**C**) show that section of the body remains unstained, almost no soft tissue contrast in the unstained area. Bottom panels show a Cinereus shrew (*Sorex cinereus cinereus* YPM:MAM:006036) with an incompletely fixed section on the left ventral side of the posterior part of the torso. The specimen's appearance before staining (**D**) shows that section of the body and its loss of hair, and the 3D rendering (**E**) and cross section through the body (**F**) show that section of the body remains unstained, and almost no soft tissue contrast in the unstained area.

As well as showing scans that can be successfully used to study anatomy, we provide examples of specimens with poorly preserved soft tissues, and the appearance of the tomograms in those scans. We provide examples of specimens with widespread tissue damage across the entirety of the specimen (Fig. 8). Tissue damage makes these tissues appear fractured and shrunken, and has inconsistent GSVs across similar tissues with some areas exhibiting high GSV (making them appear bright). These scans can often be successfully rendered in 3D (see 3D renderings in Fig. 8), while the tissue damage is evident in the appearance of the cross-sections. We also provide examples of specimens that show signs of incomplete fixing, with a portion of the body unable to take up iodine despite being in a staining solution for enough time to allow the rest of the specimen to be fully stained (Fig. 9). The parts of the body that remained unstained were observed to be less firm than the parts of the body that stained normally, suggesting these areas were not adequately fixed; additionally, prior to staining, the mammal had lost hair in the area that remained unstained. These specimens were bathed in iodine for longer than required to determine whether a longer staining time would eventually lead to staining of the poorly fixed portions, but those portions still remained unstained.

### Physical effects

We did observe some physical effects on the appearance of specimens that have been stained with iodine solution. We noticed some pigment loss in fish, amphibian, and reptile specimens. This pigment loss seemed to be more pronounced when using the buffered Lugol's iodine. All of the mammals in this study were stained using 1.25% w/v buffered Lugol's iodine (i.e., no unbuffered iodine was used on mammals). For many of the mammal specimens, we noticed some depilation during the de-staining process, with some specimens even losing all of their hair after destaining (see Supplemental File 3).

### DiceCT and birds

In this study, we avoided staining a large number of birds because of the report that bones became flexible in both water and ethanol-based iodine solutions, and that the flexibility may be due to the acidity of the staining agents used, causing demineralization of bone (Early et al. 2020). Since Dawood et al. (2021) demonstrated that adding a buffer to the iodine solution ensures the pH of that solution remains stable during staining, we stained one bird (a ruby-throated hummingbird [*Archilochus colubris* UF:Birds:52652]) in a buffered iodine solution to determine whether this could help to avoid bone demineralization. While the pH of the buffered iodine solution remained stable, the bones of the hummingbird were flexible after staining, suggesting that keeping the pH stable does not prevent bone demineralization in birds. However, we observed that the bones of the hummingbird did not become flexible during staining, but rather during destaining, indicating that the removal of iodine from the soft tissues may be the cause of the flexibility observed in the bones. It is possible that long-term storage in ethanol may contribute to the demineralization of bone, but the short time frame in which flexibility appeared in the hummingbird bones suggests that long-term ethanol storage is not a major contributing factor. Along with our observation of a stable pH, Kolmann and Nagesan et al. (2023) also observed that the decline in pH of ethylic iodine solutions during staining was nowhere near the levels required to dissolve bone tissue. Collectively, these results suggest that changes in pH are not the cause of bone demineralization during the iodine staining process. Despite the effects on the bones, we were still able to produce a high-quality (TCS = 4) diceCT scan of the ruby-throated hummingbird, which is available on MorphoSource (https://www.morphosource.org/concern/media/000576494).

### Discussion

With a focus on fluid-preserved natural history specimens, we give a broad review of the diceCT process for a wide range of vertebrates that were imaged for the oVert Thematic Collections Network (Blackburn et al. 2024). This diceCT effort is the broadest conducted so far, both taxonomically and in the diversity of body shapes and sizes. Our results and observations supplement current knowledge about diceCT protocols for natural history specimens, and we demonstrate examples of the utility of high-quality diceCT scans.

#### Considerations for future diceCT staining of natural history specimens

Unsurprisingly for a diffusion-based process, we found that staining time increases with body size and depends on other physical properties of the specimens. In general, amphibians and reptiles of a given body size stain faster than fishes and mammals of the same body size, which is likely due to anatomical differences such as the amount and distribution of muscle mass and fat content. Through monitoring the staining progress through test scans, we observed that for fishes, parts of the mid-body that are made up mostly of muscle, took the longest time to stain. For mammals, the neck and shoulders often took the longest time to stain, and these areas are also typically composed primarily of muscle. These findings are reflected in the predictive equations we provide for estimating iodine-staining times. However, we encourage researchers to investigate the anatomy of their chosen taxonomic group, and refer to our observations for each group. Knowing the anatomy of the taxon of interest will help to predict the iodine uptake of specimens.

#### Facilitating faster diffusion

The rate of diffusion can be sped up by increasing the iodine concentration of the staining solution. In this study, we used a relatively low concentration of 1.25% w/v Lugol's solution for most staining solutions. We chose to use a low solute concentration to reduce the risk of osmotic shock to delicate tissues (Gignac et al. 2016). Increasing the solute concentration within the appropriate range will increase the concentration gradient and speed up the rate of diffusion, and staining can be completed faster (Costello et al. 2024). For example, if you need to stain an 18 g fish specimen, you will estimate that it will take 47 days to stain with a 1.25% w/v solution (as in Table 1). If you need the scan in less than 47 days, then you may consider using a higher solute concentration of 2.5% w/v, and staining will be completed more quickly. This approach may be taken for relatively large specimens, or when data collection is more urgent. We recommend keeping the solute concentration at or below 5% w/v. Refreshing staining solutions more often will also facilitate faster staining times. In this study, the staining solution was refreshed when it became visibly paler. A paler solution indicates a decrease in iodine concentration and therefore a decrease in concentration gradient between the solution and the specimen, which slows diffusion. If solutions are refreshed more frequently to maintain the concentration gradient, staining will be completed in less time.

#### Considering preservation history

The most important factor to consider when choosing a specimen for diceCT is preservation history. If a specimen has not been preserved correctly or tissue damage has occurred, then that specimen is likely to produce a poor quality (see Fig. 8) or an incomplete diceCT scan (see Fig. 9). Here, we have shown that if a specimen is fixed and then stored in ethanol for more than 90 years, it can still be used to produce a high-quality diceCT scan. Therefore, we recommend choosing natural history specimens with a known preservation history, and aim for those that have been euthanized and fixed immediately. We suspect that freezing specimens multiple times before fixation is one source of tissue deterioration observed in our samples. Preservation history is not typically recorded for museum specimens, including those in this study, but we still produced high-quality datasets for the majority of specimens.

#### Making choices on a case-by-case basis

We aimed to produce full-body scans of every component of the anatomy, but for researchers needing data for only a particular tissue or region (e.g., Holliday et al. 2022), a high-resolution scan containing all of the anatomy may be unnecessary and result in lower-resolution scans. In these cases, staining protocols can be optimized or shortened to focus on the structures of interest. Sometimes the structure of interest may be completely stained, and a useful scan can be completed without waiting for the entire body to stain (though other parts of the specimen may remain under-stained). For example, for lizards such as teiids and varanids, in which the hip muscles take a long time to be stained, a usable head scan can be acquired without waiting for the whole body to be completely stained. For specimens containing eggs, they may take a very long time to stain, but if the eggs are irrelevant to that study, the rest of the anatomy can be imaged without waiting for the entirety of the specimen to be stained. Researchers may also choose to exclude specimens that are visibly gravid to shorten staining times.

#### Processing diceCT scans

DiceCT scans are innately complex and typically more challenging and time-consuming to process than regular CT data (i.e., skeletal) with no contrast enhancement. For any research group, important considerations should be given to the hardware and software resources for data manipulation, 3D model building, and subsequent model processing. Approaches to processing diceCT data will differ depending on the taxonomic group, quality of the dataset, segmentation software, and preferred approaches can differ even among individual scientists. Users should also consider that CT-processing software is frequently updated, so the user interface, tools, and functions may change over time.

**Fig. 10.**
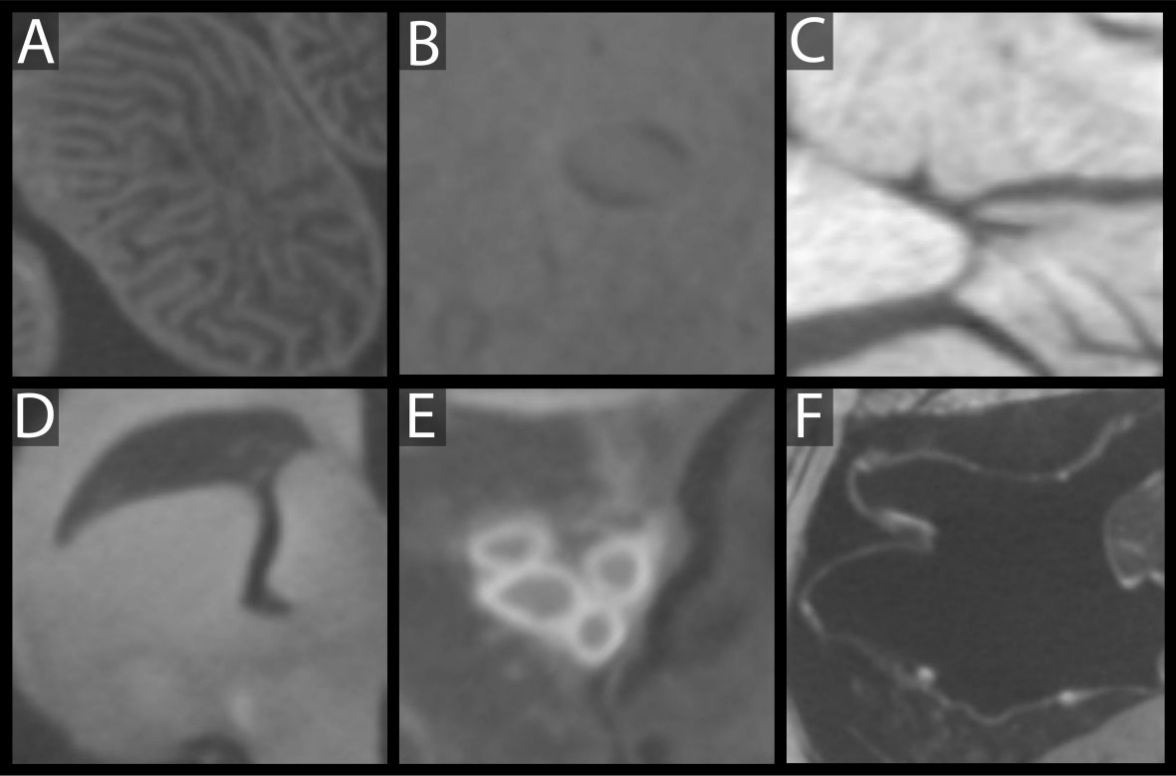
Cross sectional representations of (**A**) duodenum, (**B**) liver, (**C**) appendicular muscles, (**D**) brain, (**E**) arteries, and (**F**) lungs of a diceCT scan of *Phyllurus platyurus* (UF:herp:126970).

Although the contrast agents make the various soft tissues easier to identify and differentiate through gray-value patterns or “texture” (see Fig. 10), the similarity of gray values between soft tissue types and the contiguity of stained tissues reduce the effectiveness of standard automated segmentation techniques like thresholding or flood filling. There are several steps that can facilitate segmenting of diceCT datasets, which we outline here:

1. Reorienting the dataset so that the tomograms align to the standard orthogonal planes (coronal, sagittal and transverse) helps with navigating the dataset, facilitating the identification and delimitation of key anatomical structures. Bilaterally symmetrical structures, like nerves, muscles and the urogenital system, will appear mirrored in the standard transverse view, providing an additional point of reference when tracing these features. Where necessary, datasets can be reorientated multiple times to account for nonstandard preservation positions.
2. Tools like spherical draw (*VGStudio Max*) or paint tool (*3D Slicer*) work well for isolating specific anatomical features from diceCT datasets, especially when constrained to only select specific gray value ranges (e.g., “gray value interval” of *VGStudio Max* and “masking editable intensity range” of 3D *Slicer*). This allows the user to target key structures with subtly different gray-value ranges than their surrounding environments.
3. When isolating complicated structures like nervous or circulatory systems, the user can try segmenting into multiple parts and then combining.
4. Distinguishing tissue types. In the circulatory system, erythrocytes are small and have a high surface-to-volume ratio, the staining of their cell membranes causes blood to show up as very dense (i.e., higher GSV) in diceCT datasets. The tunica of larger blood vessels can be distinguished from the lumen and will either show up as more or less dense, depending on the presence of blood. Nerves by comparison are slightly less dense (i.e., lower GSV) and appear more uniform in cross-section. As both nerves and blood vessels appear as circular in cross section, the smaller structures can be difficult to distinguish from one another. In this case, it is often easier to trace outwards from more identifiable features (e.g., spinal cord or arteries).

### Data manipulation and digital segmentation

#### Software

There are several software tools available for processing volumetric CT data. Free options include *3D Slicer* (open-source, [Buser et al. 2020]) or *ORS Dragonfly* (Comet Technologies, available for free with an educational affiliation). For commercially available software, we recommend *VGStudio Max* (Volume Graphics) or *Avizo/Amira* (Thermo-Fisher). These platforms allow volumetric CT data to be visualized and manipulated as 3D objects, and also allow users to isolate “regions of interest” (ROIs) from their parent volumes, a process also known as “segmentation.” Each platform has a set of tools that will allow the user to select voxels manually or through semi-automated means, by choosing ranges of voxels with specific gray-scale values. ROIs can be used to generate 3D models as surface/mesh files such as stereolithography (STL), polygon (PLY), object (OBJ) format, among others. After 3D model generation, the resulting files can be edited and manipulated in mesh-editing software, of which there are many free options available. We typically recommend *Meshlab* for basic mesh editing (e.g., whole-mesh decimation, file format conversion), and *Blender* for more advanced mesh editing (e.g., finer editing, re-positioning of elements, animations). More complex statistical analyses such as geometric morphometrics (Adams and Castillo 2013) and muscle fiber tracking (Arbour 2023) can be carried out using the R statistical environment (Grunsky 2002).

#### Hardware

Processing volumetric data is a computationally demanding task and no segmentation software has the capacity to handle large scan datasets without appropriate hardware. When acquiring a computer for processing CT data, users should consider the processor (CPU), system memory (RAM), graphics card (GPU), and data storage. Users can check the minimum requirements for their chosen processing software and aim to exceed the minimum requirements of CPU, RAM, GPU and data storage. The size of scans should also be considered.

### Data management

Although we have outlined data management for this study as an example, we emphasize that data management plans will differ depending on the study and the researchers’ institutional affiliation, which typically governs their access to resources. Those hoping to develop a data management plan for their own diceCT studies should consider the technological resources and budgets offered by their home institution or funding agency, and the longevity of those resources. It may also be helpful to refer to data management plans for other types of image data (e.g., Brainerd et al. 2017). Data producers will need to make decisions regarding data classification, technical requirements, data standards, data accessibility/preservation, and compliance/oversight. The Non-Clinical Tomography Users Research Network (NoCTURN) offers a Computed tomography Dataset Management and Sharing Plan that follows U.S. guidelines on data management, available via the NoCTURN website (https://nocturnetwork.org/resources/open-science-resources/).

### Future directions

While we were able to identify and demonstrate the appearance of poorly preserved soft tissues in diceCT scans, the lack of detail recorded about preservation history for most natural history specimens makes it impossible to know how that soft tissue damage was caused. To provide more distinct information about the effects of preservation history on soft tissues, the field of diceCT would benefit greatly from studying the effects of delayed fixation (i.e., not fixing specimens immediately after death), repeated freeze/thaw cycles, incomplete fixation, suboptimal storage, and other potential events that could affect soft tissue preservation. Future experiments could also expand on the work of Gignac et al. (2024) to investigate whether the combination of exposure to both Lugol's iodine and X-rays impairs DNA quality in preserved samples. Additionally, more work could be done to examine how shrinkage may affect the volume of soft tissues in natural history specimens.

### Conclusions

We provided results, observations, and recommendations for using diceCT to generate high-resolution 3D anatomical scans, which can be easily shared digitally with collaborators, educators, and the general public. Additionally, data produced during this study will remain available for use in future research, education, art, or other projects. We hope that the publication and availability of these scans on MorphoSource will facilitate future exploration of the soft tissue anatomy of vertebrates.

## Supplementary Material

obaf014_Supplemental_Files

## Data Availability

Fish: https://www.morphosource.org/projects/000598946 Amphibians: https://www.morphosource.org/projects/000600196 Reptiles: https://www.morphosource.org/projects/000602028 Mammals: https://www.morphosource.org/projects/000604886 Ruby-throated hummingbird: https://www.morphosource.org/concern/media/000576494
