# Supplementary material for: The Diffusion Diaries: Diffusible Iodine-Based Contrast-Enhanced Computed Tomography for Vertebrate Natural History Specimens
Source: Integr Org Biol. 2025 Apr 7;7(1):obaf014. doi: 10.1093/iob/obaf014 (PMC12010875; doi:10.1093/iob/obaf014)
Supplement: obaf014_Supplemental_Files [file obaf014_supplemental_files.zip › Supplementary file 1 - oVert diceCT protocol.pdf]

oVert DiceCT Wet Lab Protocol

1

STANDARD CT SCAN + PRE-SCAN DATA COLLECTION

2

WALK DOWN ETOH CONCENTRATION

2-3 days for each step down

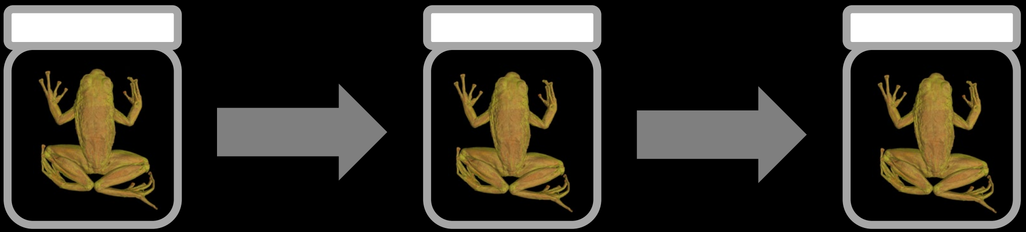

70%

Museum specimens are stored in 70% EtOH

50%

To make 50% EtOH
Add 40 ml H_2O for every 100 ml 70% EtOH

30%

To make 30% EtOH
Add 130 ml H_2O for every 100 ml 70% EtOH

3

STAIN WITH LUGOL'S IODINE SOLUTION

Prepare a stock solution of 15% Lugol's iodine

5 g of I_2 and 10 g of KI, made up to 100 ml de-ionized H_2O.

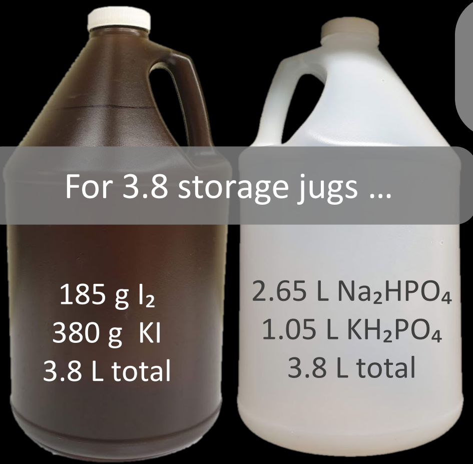

Prepare a 2x Sorensen's buffer

For 266 mM Na_2HPO_4
Mix 37.76 g Na_2HPO_4 made up to 1 L de-ionized H_2O

For 266 mM KH_2PO_4
Mi 36.20 g KH_2PO_4 made up to 1 L de-ionized H_2O

Test for pH = 7.2

Prepare buffered Lugol's solution of target concentration

Target %	Volume of 15% Lugol's	Volume of DI H_2O	Volume of buffer	Total volume
1.25	8.33	41.67	50	100
2.50	16.67	33.33	50	100
3.75	25	25	50	100

OR

Prepare Lugol's solution of target concentration

Target %	Volume of 15% Lugol's	Volume of DI H_2O	Total volume
1.25	8.33	91.67	100
2.50	16.67	83.33	100
3.75	25	75	100

Sigma-Aldrich product numbers →
I_2: 207772 **Na_2HPO_4:** S0876
KI: 221945 **KH_2PO_4:** P9791

Submerge specimen in prepared solution

1.25% B-I_2KI or I_2KI

for most specimens

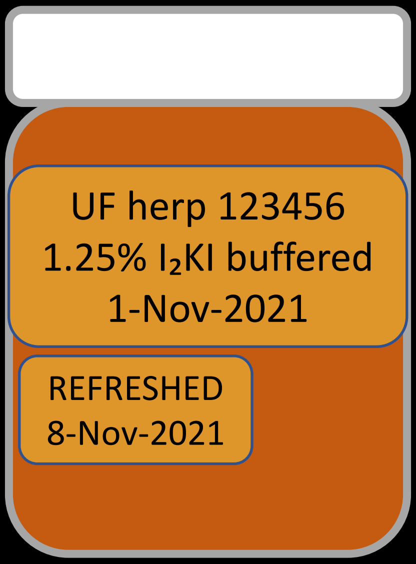

2.5% or 3.75% B-I_2KI or I_2KI

for large or difficult specimens

Choose a staining vessel with plenty of room for enough staining solution. If possible, vessel should seem "too big" for specimen.

Label each staining vessel with

- Specimen number
- Staining solution
- Date in stain
- New label with date for each solution refresh

Keep an eye on solution as staining progresses and refresh solution if it starts to look pale.

4

ASSESS STAIN PROGRESSION

When specimen has been in solution for sufficient time, conduct 5-10 min rapid scan to assess staining progression. If iodine diffusion gradient is visible (see images), return to staining vessel and refresh solution.

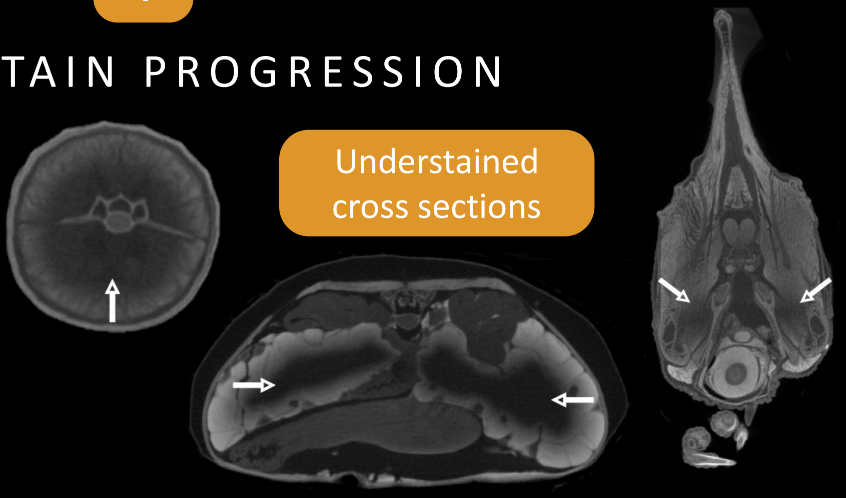

5

PERFORM DIFFERENTIATION

Removes unbound iodine and helps even out overstained surfaces
Submerge specimen in 30% EtOH bath before scanning

Amphibians	Reptiles	Specimens with overstained extremities
~ 30 minutes	*~ 60 minutes*	*> 1 hour*

Specimen with excessive overstaining can be left in EtOH bath for up to 24 hours

Step 6

DICECT SCANNING

Packing – try to pack specimen so that external surfaces are not touching, and use low density foam to separate parts of the body.

Scanning – increase voltage, current, and filtering for stained specimens.

Step 7

DESTAINING

Walk the EtOH concentration back up
2-3 days for each step up

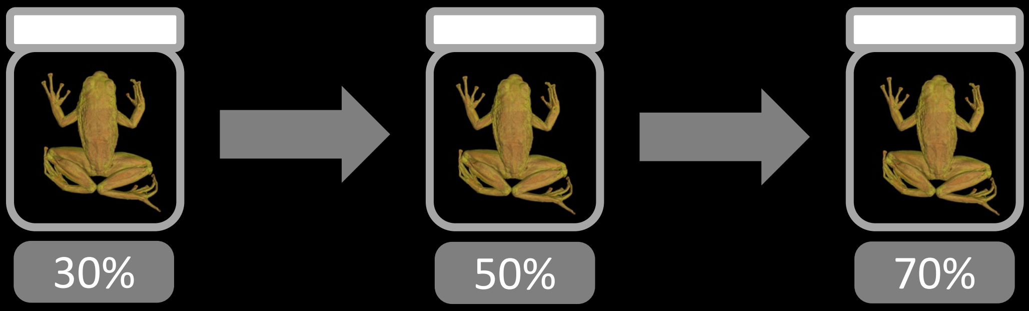

Leave in 70% EtOH

Refresh the solution regularly until destaining is complete. Iodine will leach back out of specimen, and larger specimens will take longer to destain. Destaining is complete when EtOH remains completely clear.
