# Supplementary material for: The Diffusion Diaries: Diffusible Iodine-Based Contrast-Enhanced Computed Tomography for Vertebrate Natural History Specimens
Source: Integr Org Biol. 2025 Apr 7;7(1):obaf014. doi: 10.1093/iob/obaf014 (PMC12010875; doi:10.1093/iob/obaf014)
Supplement: obaf014_Supplemental_Files [file obaf014_supplemental_files.zip › Supplementary file 2 - DiceCT scoring guidance.docx]

**Tissue Condition Score (TCS): guidance for scoring**

1: very low-quality soft tissues, with widespread damage and very little or no usable anatomical structures detected

2: somewhat low-quality soft tissues, with substantial tissue damage but some usable anatomical structures

3: mostly good quality soft tissues, with some tissue damage detected but most anatomical structures usable

4: very good quality soft tissues, no tissue damage detected and all anatomical structures usable

Below, we suggest characteristics to consider when deciding on the quality and usefulness of the soft tissues in a diceCT dataset. While no single one of these characteristics dictate the overall TCS, the ability of users to isolate anatomical structures in the dataset should be considered when scoring.

| Character | Low quality | High quality |
| --- | --- | --- |
| Texture | - Anatomical structures can appear fragmented or porous in appearance. - Often with uneven stain uptake that can make structures appear “blotchy”. - Anatomical structures may have rough/irregular boundaries. - See figure 8. | - Anatomical structures appear smooth and uniform in appearance. - Stain uptake appears even with uniform grayscale values across anatomical structures. - Anatomical structures have smooth and well-defined boundaries. |
| Shrinkage | - Structures have separated from each other within the body of the animal, with large gaps in between organs, between muscles, and sometimes between soft tissue and bone (a typical sign is a large separation between the brain and the braincase). - Visible areas that appear oversaturated with iodine and “washed out”, e.g. a muscle that has very high grayscale values and no visible fibers. - Typically symmetrical structures (e.g. the brain) can display some degree of asymmetry or appearance of “slumping” in a particular direction. | - Margins of structures remain in place with no large spaces introduced between them. - No oversaturation of iodine and good amount of contrast between high and low density structures. - Structures appear symmetrical with no sign of slumping. |
| Physical condition (observe prior to imaging) | - Specimen has been dissected previously, with visible cuts or removal of tissue. | - Specimen is completely intact, with no visible cuts or removal of tissue. |
| Quality of fixation | - Patches of specimen remain under stained or unstained. - See figure 9. | - Specimen is evenly stained throughout entire body. |
