# Supplementary material for: The Diffusion Diaries: Diffusible Iodine-Based Contrast-Enhanced Computed Tomography for Vertebrate Natural History Specimens
Source: Integr Org Biol. 2025 Apr 7;7(1):obaf014. doi: 10.1093/iob/obaf014 (PMC12010875; doi:10.1093/iob/obaf014)
Supplement: obaf014_Supplemental_Files [file obaf014_supplemental_files.zip › Supplementary file 3 - mammal depilation.pdf]

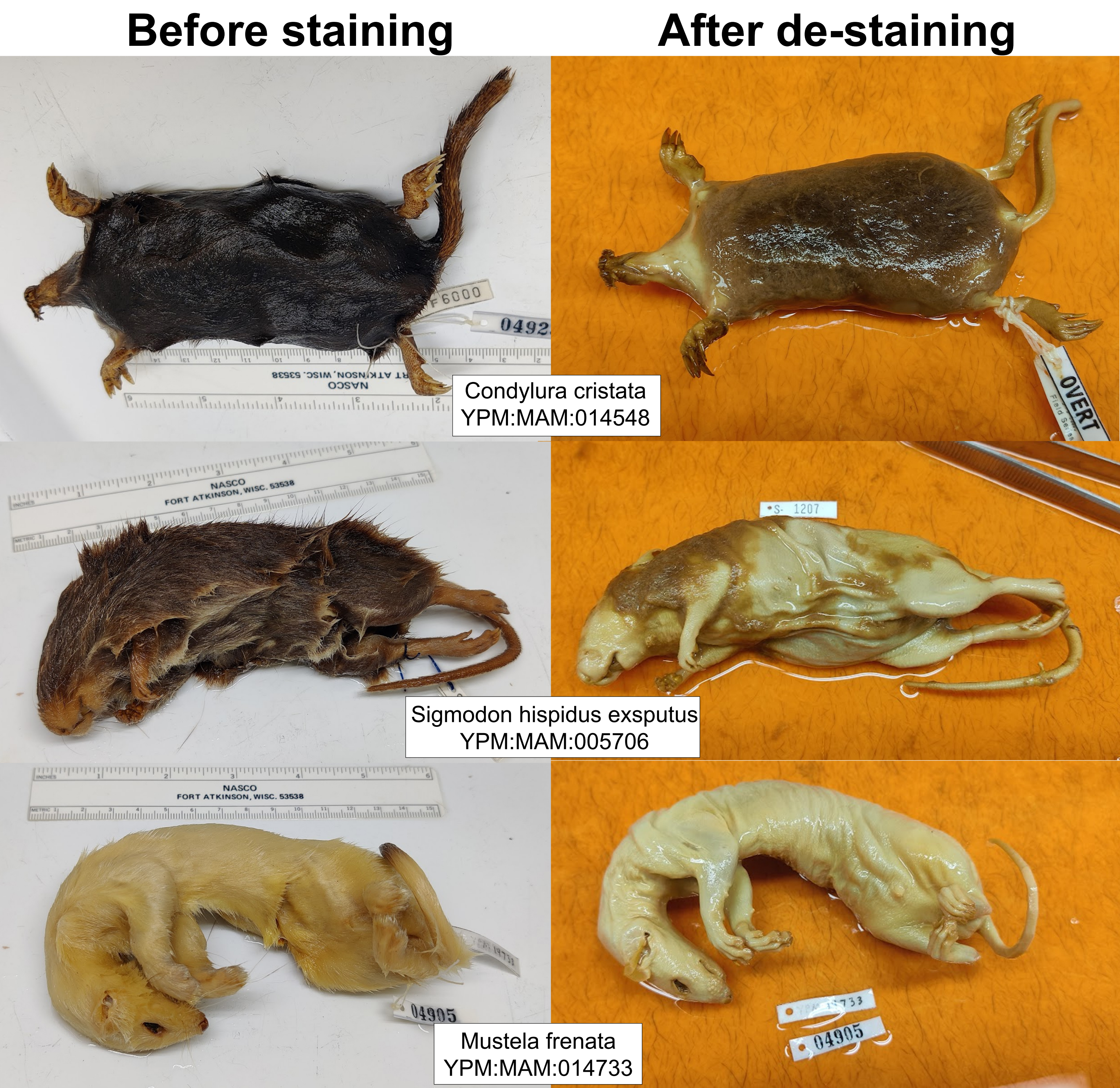
Before staining
After de-staining
Condylura cristata
YPM:MAM:014548
Sigmodon hispidus exsputus
YPM:MAM:005706
Mustela frenata
YPM:MAM:014733
NASCO
FORT ATKINSON, WISC. 53538
F6000
S- 1207
04905
